# Psychological readiness is related to return to sport in judo injuries: a cross-sectional study

**DOI:** 10.1186/s13102-023-00631-5

**Published:** 2023-02-16

**Authors:** Christophe Lambert, Daniel Guenther, Lisa-Marie Schütz, Niklas Kern, Ramona Ritzmann, Noémie Reinert, Martin Walz, Arasch Wafaisade, Kolos Nagy, Sven Reuter

**Affiliations:** 1 Department of Trauma and Orthopedic Surgery, University of Witten/Herdecke, Cologne Merheim Medical Centre, Ostmerheimer Str. 200, 51109 Cologne, Germany; 2 Institute of Sports and Sports Sciences, Heidelberg University, Heidelberg, Germany; 3 SRH Hochschule Für Gesundheit Gera, Campus Stuttgart, Gera, Germany; 4 Department of Sports and Sports Sciences, University of Freiburg, Freiburg, Germany; 5 Department of Orthopedics and Traumatology, University Hospital Basel, Basel, Switzerland; 6 TUM Department of Sport and Health Sciences, Technical University of Munich, Munich, Germany; 7 Southern Methodist University, Dallas, USA

**Keywords:** Psychological readiness, Elite sports, Athletes, Injury, Knee, Shoulder, Mental health

## Abstract

**Background:**

The aim of this study is to investigate the influence of a judoka's psychological readiness in relation to his ability to return to sport. At the present time, the relationship between physical and psychological readiness to return to sport has not been adequately elucidated.

**Methods:**

This is a cross-sectional study. An online survey was distributed via social networks and the German Judo Association collecting data from competitive and recreational judo athletes. The survey collected data on participants’ characteristics, history of injury, and psychological readiness to return to sport after injury as determined by either the Anterior Cruciate Ligament-Return to Sport after Injury Scale, the Shoulder Instability-Return to Sport after Injury Scale, or a modified version of the Return to Sport after Injury Scale depending on the respective type of injury.

**Results:**

The study included 383 judo athletes (272 competitive judo athletes and 112 recreational judo athletes). Regardless of injury location, athletes who achieved return to sports (*M* = 70.67; *SD* = 16.47) had higher RSI scores than athletes that did not return to sports (*M* = 53.88; *SD* = 19.12; *p* < 0.0001). Male athletes (*M* = 65.60; *SD* = 19.34) did show significantly higher RSI scores than female athletes (*M* = 60.45; *SD* = 19.46). The RSI score differed for different time loss categories, *F*(7, 375) = 11.309*, p* < 0.001, *η*^2^ = .174 with decreasing RSI scores for longer time loss and lowest RSI scores in athletes, who never returned to sports. RSI scores of athletes with knee injuries differed from athletes with other injury locations (10.23, 95% CI [4.08, 16.38]). After adjusting for time loss due to injury, competitive athletes had higher RSI scores than competitive athletes (*F* (1, 382) = 7.250, *p* < 0.001, partial *η*^2^ = .02). Conservatively treated athletes (*M* = 66.58; *SD* = 18.54) had higher RSI scores than surgically treated athletes (*M* = 59.05; *SD* = 20.01; *p* < 0.05).

**Conclusion:**

Based on the data of this study, type of injury, sport level, treatment method, and gender appear to influence psychological readiness on judoka and their ability to return to sport. The multiple factors that influence a judoka and their ability to return to sport argue for individualized treatment of judoka and their psychological state after injury in the return to sport process.

## Introduction

The effect of mental health and the influence of various psychological aspects on recovery from injury has only recently moved into the focus of elite sports [1]. A sports injury represents a critical life event and has the potential to induce enormous stress within the affected individual [2]. Critical life events, especially those eliciting chronic stress, can induce adverse physiological changes and can thus increase injury rate [3, 4]. Typical stress reactions in the acute phase of an injury may include negative self-talk, negative emotions such as anxiety and depression, psychosomatic complaints such as fatigue, or stress reactions such as loneliness, which includes feelings of social isolation [5]. The psychological stress response of athletes depends on numerous factors, including the severity of the injury, the performance level of the athlete, situational factors, and setbacks encountered in the course of treatment [6, 7].

Among Olympic disciplines, elite Judo has, one of the highest injury incidences. Kujala et al. found considerably higher injury rates in judo and karate than in conventional team sports such as soccer, ice hockey, volleyball, or basketball [8]. During the 2008 and 2021 Olympic Games, 11–12% of individuals competing in judo suffered an injury. In addition to minor knee, finger, and shoulder injuries, there is the potential for serious injuries that can lead to prolonged downtime of months or even years [9, 10]. The most common and severe injury, in terms of duration of recovery and loss of performance, involves the knee and shoulder [10].

A current consensus statement for return to sport advocates to use comprehensive tests that includes not only a physical, but also psychological screening of the athletes [11]. The authors of the consensus statement propose the “Anterior Cruciate Ligament Return to Sport after Injury” (ACL-RSI) scale to assess the psychological readiness of athletes after injury [12, 13]. The scale has also been validated for other parts of the human body [14–16]. In addition to these scales, several other factors such as gender, age, and performance level play a key role in the incidence of injuries in judokas [17].

The purpose of this study is to assess the psychological aspect of a judoka's return to sport using the ACL-RSI scale. Our goal is to shed light on external and internal psychological factors that may influence an athlete’s psyche and return to sport. We hypothesize that athletes who return to sport differ in their psychological readiness from athletes that do not return to sport based on gender, level of performance, and the injury-related time loss.

## Material and methods

### Procedures

The research team used the “Declaration of Helsinki ethical principles for medical research involving human subjects” as the standard. Inclusion criteria for the study consisted of (1) competitive or recreational judo athletes with age 18 and above; (2) proficiency in the English or German language; (3) written consent to the privacy policy statement; and (4) having suffered an injury within the last 5 years. Exclusion criteria was (1) an incomplete questionnaire.

The survey consisted of a total of 23 items and was presented in the format of an online questionnaire. The survey was distributed by international judo federations, it was open for 60 days, from September to October of 2020.

### Instruments

An online survey was distributed via social networks and the German Judo Association collecting data from competitive and recreational judo athletes. The German Judo Association sent the link as an email to all national team athletes and asked them to fill it out. The survey collected data on participants’ characteristics, history of injury, and psychological readiness to return to sport after injury as determined by either the Anterior Cruciate Ligament-Return to Sport after Injury (ACL-RSI) Scale, the Shoulder Instability-Return to Sport after Injury (SI-RSI) Scale, or a Modified version of the Return to Sport after Injury (M-RSI) Scale depending on the respective type of injury.

The survey comprised of four sub-sections which considered the site of injury and the athlete’s injury history. The survey contained questions about the athlete’s feelings towards their sporting activity and their return to sport. Athletes were categorized into “return to sport” if they answered “higher level” or “same level” when asked about which level of performance they achieved after injury. Athletes who answered the question with “reduced level” or “stop judo” were categorized in “no return to sport”.

#### Participant characteristics

The first section of the questionnaire included five general questions about the participants` characteristics. Participants were asked about their gender, age, nationality, weight class, and their performance level (international, national, regional or recreational level).

#### Injury history

The second section included 6 questions. The questions covered the injured joint, injured structure(s), the precise medical nomenclature of the injury, the therapy (*surgical or conservative*), the down time after injury, and the level of performance achieved after having finalized the therapy. Participants who experienced an injury in the last 5 years proceeded with the surveys. Injuries were distinguished by *location*: knee, shoulder, both, and other. Most important injuries have been conserved as follows: Anterior cruciate ligament rupture, medial collateral ligament rupture, lateral collateral ligament rupture, and acromioclavicular joint dislocation.

#### Psychological readiness to return to sport after injury

*Anterior Cruciate Ligament–Return to Sport after Injury questionnaire (ACL-RSI)* The validated version of the ACL-RSI scale includes 12 questions pertaining to the athlete’s emotional well-being and their confidence in his or her performance and subsequent risk appraisal. The scale gives an indication of the psychological readiness after severe injury to return to sport [18]. The scale, with scores ranging from 1 to 10, includes 5 questions on emotional well-being, 5 questions on confidence in physical performance, and 2 questions on the risk appraisal. Higher scores indicated a more positive psychological response, thus a higher psychological readiness. The total score was determined by adding the values of the 12 responses and then calculating their relationship to 100 to obtain a percentage.

*The Shoulder Instability-Return to Sport after Injury (SI-RSI)* The SI-RSI questionnaire was chosen as a valid and reproducible scale to quantify psychological readiness to return to sport after traumatic shoulder instability [15]. The structure of the survey, the number of questions, and the procedure for calculating the scores are the same as those used in the ACL-RSI questionnaire as described above.

*Modified version of the Return to Sport after Injury (M-RSI) Scale* M-RSI was used when athletes reported other injuries than knee and shoulder. All questions remained the same, only the location of the injury was generalized. For example, "knee" was replaced by "body" in the relevant items. It has been shown that the ACL-RSI can be modified to other regions of the body being a valid tool for the assessment of psychological readiness to return to sport [14].

#### Time loss due to injury

Time loss due to injury was assessed via self-report. Participants indicated whether injury lasted less than 3 weeks (7), 3–6 weeks (6), 6–12 weeks (5), 3–6 months (4), 6–9 months (3), 9–12 months (2), longer than 12 months (1) or still not returned to sport (0).

#### Level of performance

Level of performance was assessed via self-report. Participants indicated whether they were performing on a recreational or competitive level (regional competitions and higher).

### Statistics

Statistical analyses were performed using SPSS 27 (Statistical Package for Social Science, IBM^®^). An independent* t*-test was calculated to determine statistical differences in RSI scores (ACL-RSI, SI-RSI and M-RSI, respectively) between subjects with a successful return to sport as compared to subjects without return to sport according to our hypothesis.

In additional analyses using independent t-tests statistical effects of performance level (recreational vs. competitive), injury treatment (surgical vs. conservative), and gender (male vs. female) on RSI scores were calculated. To analyze possible differences on RSI scores comparing injury location (knee, shoulder, both, and other) an ANOVA was calculated.

Additionally binomial logistic regressions were performed to statistically investigate whether time loss due to injury, RSI scores, and other relevant variables predict return to sport. The level of significance was set at *p* < 0.05.

## Results

**Fig. 1:**
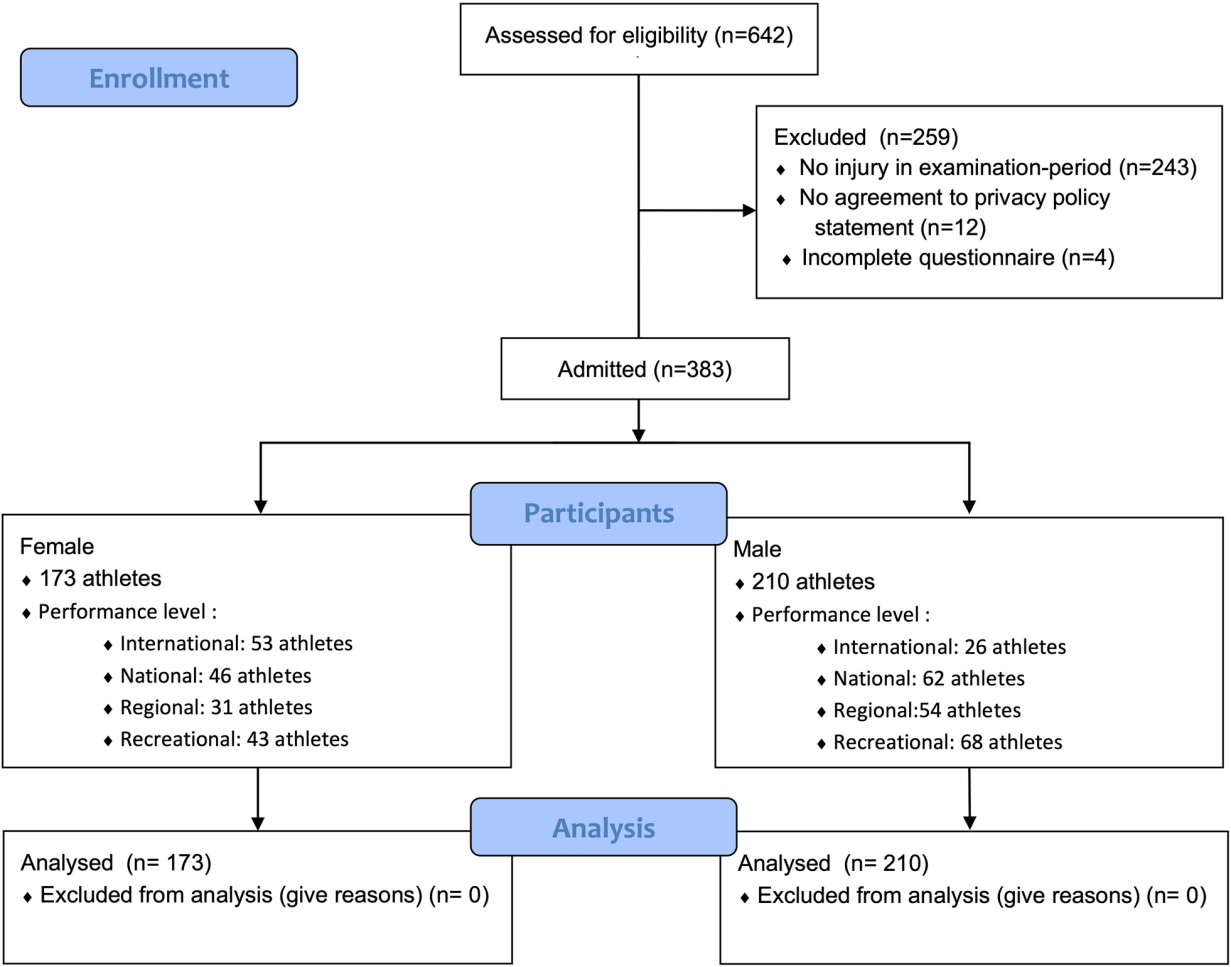
Flow chart of the enrollment, participation, and data analysis phases from top to bottom

A total of 641 athletes participated in the survey. 12 participants were excluded because they had not signed the written consent, and 7 were excluded due to an incomplete questionnaire. Of the remaining 625 athletes, 258 were excluded because they had not sustained an injury during the study period. A total of 383 were included in the final analysis (Fig. 1).

A total of 55.9% (*n* = 214) of the examined athletes reported a return to sport and 44.1% (*n* = 169) reported no return to sport. To analyze the difference between athletes with return to sport and without, a *t*-test was calculated. Results confirm the hypothesis, that athletes that return to sport differ in their psychological readiness from athletes that do not, (381) = 9.22, *p* < 0.001, *d* = 0.95, 95% CI [0.74, 1.16]. Athletes that return to sport (*M* = 70.67; *SD* = 16.47) had significantly higher scores than athletes that did not return to sport (*M* = 53.88; *SD* = 19.12).

### Influence of treatment method

169 athletes (44.1%) underwent surgery as part of their treatment with a 43.2% return to sport rate (*n* = 73). 214 athletes (55.9%) underwent non-surgical treatment, with a return to sport rate of 65.9% (*n* = 141). When looking at the RSI scores, an independent *t*-test revealed, that conservatively treated athletes differed from surgically treated athletes,* t*(381) = 3.81, *p* < 0.001, *d* = 0.39, 95% CI [0.19, 0.56]. Conservatively treated athletes (*M* = 66.58; *SD* = 18.54) had significantly higher RSI scores than surgically treated athletes (*M* = 59.05; *SD* = 20.01).

### Influence of gender

Of 209 male athletes, 118 returned to sport (56.5%), whereas 91 (43.5%) did not. Among female athletes, 96 (55.2%) of 174 athletes returned to sport, whereas 78 (44.8%) did not. Gender differences were found regarding RSI scores, *t*(381) = 2.59, *p* = 0.010, *d* = 0.27, 95% CI [0.06, 0.47]. Male athletes (*M* = 65.60; *SD* = 19.34) did show significantly higher RSI scores than female athletes (*M* = 60.45; *SD* = 19.46).

### Influence of injury location

Of 130 athletes with knee injuries, 63 returned to sport (48.5%) whereas 67 did not (51.5%). Among athletes with shoulder injuries, 53 (55.8%) out of 95 did return to sport, whereas 42 (44.2%) did not. Of 30 athletes with both knee and shoulder injuries, 19 returned to sport (63.3%) whereas 11 did not (36.7%). Among athletes with other injury locations, 79 (61.7%) out of 128 returned to sport, whereas 49 (38.3%) did not.

We conducted a one-way ANOVA to assess the effects of injury location on RSI score. Injury location was divided into four categories: knee (*M* = 58.26, *SD* = 19.54), shoulder (*M* = 63.87, *SD* = 19.73), both knee and shoulder (*M* = 61.52, *SD* = 14.64) and other (*M* = 68.39, *SD* = 17.62). The RSI score differed statistically significant for the different injury location, *F*(3, 379) = 6.24, *p* < 0.001, η^2^ = 0.05. Scheffé post-hoc analysis revealed a significant difference (*p* < 0.001) between RSI scores of athletes with knee and other injury locations (10.23, 95% CI [4.08, 16.38]).

Further a binomial logistic regression was calculated to determine the effect of RSI score and injury location to predict the likelihood of return to sport. The binomial logistic regression model was statistically significant, χ^2^(3) = 75.10, p < 0.001, resulting in a small amount of explained variance Nagelkerke’s *R*^2^ = 0.239. RSI score (*p* < 0.001) significantly predicted return to sport, while injury location (*p* = 0.85) and the interaction (*p* = 0.89) showed no significant effect.

### Influence of performance level

A total of 108 recreational and 275 competitive athletes participated in this study. 67.6% (*n* = 73) of recreational athletes reached return to sport compared to 51.3% (*n* = 141) of competitive athletes. When looking at the RSI scores, an independent *t*-test revealed, that recreational athletes (*M* = 63.52 *SD* = 21.42) do not differ regarding their RSI score, compared to competitive athletes (*M* = 63.15; *SD* = 18.79), *t*(381) = − 0.17, *p* = 0.087, *d* = − 0.02, 95% CI [− 0.24, 0.20].

We further performed a one-way ANOVA to analyze the influence of the performance level on RSI score after adjusting for time loss due to injury. After adjusting for time loss due to injury, RSI scores differed for the different levels of performance (recreational vs. competitive), *F*(1, 382) = 7.250, *p* < 0.001, partial η^2^ = 0.02.

### Influence of time loss due to injury

5 athletes reported time loss due to injury of less than 3 weeks, with a positive return to sport rate of 100%. 65 athletes reported time loss due to injury of 3–6 weeks (return to sport rate = 86.2% [n = 56]), 74 athletes of 6–12 weeks (return to sport rate = 64.9% [*n* = 48]), 82 athletes of 3–6 months (return to sport rate = 51.2% [*n* = 42]), 64 athletes of 6–9 months (return to sport rate = 48.4% [*n* = 31]), 40 athletes of 9–12 months (return to sport rate = 55.0% [*n* = 22]), 31 athletes of longer than 12 months (return to sport rate = 32.3% [*n* = 10]) and 22 athletes were still not returned to sport.

We conducted a one-way ANOVA to assess the general effects of time loss due to injury on RSI score. Time loss due to injury was divided into seven categories: less than 3 weeks (*M* = 80.17, *SD* = 11.23), 3–6 weeks (*M* = 73.77, *SD* = 18.23), 6–12 weeks (*M* = 67.02, *SD* = 16.68), 3–6 months (*M* = 65.18, *SD* = 18.94), 6–9 months (*M* = 56.42, *SD* = 17.51), 9–12 months (*M* = 62.60, *SD* = 18.94), longer than 12 months (*M* = 54.65, *SD* = 20.15) or still not returned to sport (*M* = 41.78, *SD* = 13.40). The RSI score differed for the different time loss categories, *F*(7, 375) = 11.309, *p* < 0.001, η^2^ = 0.174. Scheffé post-hoc analysis revealed a difference between RSI scores of athletes that still not returned to sport and athletes with time loss of 9–12 months (*p* = 0.009; − 20.82, 95% CI [− 38.77, − 2.89]), 3–6 months (*p* < 0.001; − 23.40, 95% CI [− 39.64, − 7.12]), 6–12 weeks (*p* < 0.001; − 25.24, 95% CI [− 41.65, − 8.82]), 3–6 weeks (*p* < 0.001; − 31.99, 95% CI [− 48.67, − 15.31]) less than 3 weeks (*p* = 0.01; − 38.39, 95% CI [− 71.88, − 4.89]). Further Scheffé post-hoc analysis revealed a difference between RSI scores of athletes with time loss longer than 12 months and athletes with time loss of 3–6 weeks (*p* = 0.001; − 19.11, 95% CI [− 33.88, − 4.36]).

A binomial logistic regression was calculated to determine the effect of RSI score and time loss due to injury to predict the likelihood of return to sport. The binomial logistic regression model was statistically significant, χ^2^(3) = 101.72, *p* < 0.001, resulting in a small amount of explained variance Nagelkerke’s *R*^2^ = 0.312. RSI score (*p* = 0.003) significantly predicted return to sport, whereas time loss due to injury (*p* = 0.056), and the interaction (*p* = 0.570) showed no significant effect.

The most important results of the RTS are displayed in Table 1.

**Table 1:** Influence of Different Variables on the RSI Score

Variable	*M*	*SD*
*Treatment method*		
Conservative	66.58	18.54
Surgical	59.05	20.01
*Gender*		
Male	65.60	19.34
Female	60.45	19.46
*Injury Location*		
Knee	58.26	19.54
Shoulder	63.87	19.73
Knee + shoulder	61.52	14.64
Other	68.39	17.62
*Performance Level*		
Recreational	63.52	21.42
Competitive	63.15	18.79
*Time loss due to injury*		
< 3 weeks	80.17	11.23
3–6 weeks	73.77	18.23
6–12 weeks	67.02	16.68
3–6 months	65.18	18.94
6–9 months	56.42	17.51
9–12 months	62.60	18.94
> 12 months	54.65	20.15
No RTS	41.78	13.40

## Discussion

The aim of this study was to elaborate on the influence of psychological factors and psychological readiness in relation to the return to sport rates of judokas after knee-, shoulder-, and other injuries. To address this question, athletes filled out an online questionnaire which included questions about the injury site, injury history, and feelings towards their sporting activity and return to sport.

The result show that judokas with return to sport have significantly higher RSI scores. While athletes with return to sport showed RSI scores above 70, judokas without return to sport had a mean RSI score below 55. Further RSI score significantly predicted return to sport whereas other variables such as time loss due to injury did not. Consistent with the findings of Akoto et al., injuries to the knee and the shoulder, or both were most common in this study [10]. Injuries to the knee were associated with the lowest RSI scores, which indicates a weaker probability of an athlete reaching the same level of performance as before. These scores illustrate the significance of the location of an injury on an athlete’s psyche [6]. This is consistent with the known RSI score thresholds of 60 and 65 for return to sport documented in other studies [6, 15, 16, 19]. The most serious injury regarding return to sport rate and RSI score assessment was the ACL injury; this finding is consistent with previous literature showing the most significant reduction in performance levels after suffering this injury [10].

In this study overall time loss due to injury did not predict return to sport. Olsen et al. describes severe injuries as those resulting in a time loss from sporting activity of more than 21 days [20]. In this study, judokas with a “short” period of time loss (6 weeks or less) had a return to sport rate above 90% and an RSI score above 73. These athletes achieved similar scores to uninjured athletes in a study by Phelan et al., presenting with an average RSI score of 80.17 (*SD* = 11.23) [21].

This study did not show any general differences between the RSI scores of recreational and competitive judokas, only when adjusting for time loss due to injury differences between recreational and competitive athletes. This might suggest that minor injuries have a considerably more negative affect on the mental responses of recreational athletes compared to competitive athletes, who seem to cope more effectively with the same circumstances. This data is supported by the work of Sadeqi et al., assuming that there is a better psychological response and higher RSI scores for competitive athletes [6]. In addition, there is evidence in existing literature that suggests that a higher level of athletic performance is associated with an elevated possibility of a return to sport [7]. In summary, the psychological condition of an athlete plays an equal role in competitive and non-competitive athletes alike.

Moreover, the treatment of injury (conservative vs. surgical) is associated with different return to sport rates and RSI scores. Athletes who underwent non-surgical treatment presented not only with a higher RSI score, but also reached their initial performance levels faster. When focusing on the conservative (non-surgical) treatment, athletes with and without return to sport presented with similar high mean RSI scores. These results could lead to the assumption that athletes who underwent surgery might present with a worse mental response compared to athletes who did not undergo surgical treatment. In other words, more serious injuries have a more significant impact on the psychological health of athletes. Despite the treatment, Gerometta et al. established a validated modification of the ACL-RSI score to reproducibly assess the return to sport after shoulder instability [15]. However, the study did not further evaluate group-specific differences between surgical and conservative treatments.

In literature, gender differences in terms of return to sport have been explored [22]. Several authors mention the relationship between male athletes and a better psychological response after ACL injuries [7, 13, 23]. This is supported by our study, which found significantly higher RSI scores for male compared to female judokas. This trend gains further validity in the subgroups with return to sport and without return to sport, with the most significant gender differences seen in return to sport after a time loss of more than 9 months. These findings could emphasize that compared to their male counterparts, female athletes are more prone to a negative psychological response after long periods of time away from their sport.

### Limitations

The study contains limitations that should be mentioned. First, not all participants completed a standardized, validated questionnaire. Only the participants with injuries to their knees or shoulders completed the validated version of the questionnaire [15, 18]. The remaining participants received a modified version in which their location of injury was substituted in the questions; similar approaches to this have been found in the existing literature [15, 16, 21]. Another limitation of the study is that the data was collected retrospectively, which can lead to the over or underreporting of injuries. Furthermore, in addition to national sports association websites, participants were recruited through social media outlets such as Facebook and Instagram. Although Whitaker et al. describe this as a useful tool for participant recruitment, they simultaneously point to the fact that this method does not reach every target group to a similar degree. Using social media outlets subjects the study to an overrepresentation of younger participants [24]. Self-reported data can imply a potential for over- or under-reporting of specific diagnoses. A selection bias might also exist due to the online survey as only younger people might use this tool and thus older people are less represented in the study. With regards to the relationship between RSI score and return to sport levels, the study cannot conclusively demonstrate whether a low RSI score is the reason for a lower return to sport level or if a low return to sport level is the reason for a lower RSI score. Lastly, possible factors such as re-injury or complications during the rehabilitation process were not considered. This could also lead to a bias.

## Conclusion

When assessing return to sport and the athlete’s psychological response using a modified RSI score resp. for judokas, differences in scores arise based on the athletes’ level of performance, gender, and injury location. Regardless of the type of injury, there is a clear correlation between the psychological state of an athlete and the return to sport for injuries that cause a longer time loss from sporting activity. Though psychological interventions are not part of rehabilitation treatment yet, they should be considered during the rehabilitation process. Since the study does not distinguish whether the results of the RSI score are influenced by the results of the return to sport or whether the results of the return to sport are influenced by the results of the RSI score, further studies should be conducted to analyze this relationship in a prospective setting.

## Data Availability

The datasets used and/or analyzed during the current study are available from the corresponding author on reasonable request.

## Abbreviations

ACL-RSI: Anterior cruciate ligament–return to sport after injury
SI-RSI: Shoulder instability-return to sport after injury
M-RSI: Modified-return to sport after injury
